# Microbial Degradation of Different Hydrocarbon Fuels with Mycoremediation of Volatiles

**DOI:** 10.3390/microorganisms8020163

**Published:** 2020-01-23

**Authors:** Agota Horel, Silke Schiewer

**Affiliations:** 1 Institute of Soil Sciences and Agricultural Chemistry, Centre for Agricultural Research, Herman O. St. 15, 1022 Budapest, Hungary; 2 Civil and Environmental Engineering Department, Water and Environmental Research Center, University of Alaska Fairbanks, P.O. Box 755900, Fairbanks, AK 99775-5900, USA; sschiewer@alaska.edu

**Keywords:** biodegradation, fungal remediation, biodiesel, hydrocarbon degradation

## Abstract

Naturally occurring microorganisms in soil matrices play a significant role in overall hydrocarbon contaminant removal. Bacterial and fungal degradation processes are major contributors to aerobic remediation of surface contaminants. This study investigated degradation of conventional diesel, heating diesel fuel, synthetic diesel (Syntroleum), fish biodiesel and a 20% biodiesel/diesel blend by naturally present microbial communities in laboratory microcosms under favorable environmental conditions. Visible fungal remediation was observed with Syntroleum and fish biodiesel contaminated samples, which also showed the highest total hydrocarbon mineralization (>48%) during the first 28 days of the experiment. Heating diesel and conventional diesel fuels showed the lowest total hydrocarbon mineralization with 18–23% under favorable conditions. In concurrent experiments with growth of fungi suspended on a grid in the air space above a specific fuel with little or no soil, fungi were able to survive and grow solely on volatile hydrocarbon compounds as a carbon source. These setups involved negligible bacterial degradation for all five investigated fuel types. Fungal species able to grow on specific hydrocarbon substrates were identified as belonging to the genera of *Giberella*, *Mortierella*, *Fusarium*, *Trichoderma*, and *Penicillium*.

## 1. Introduction

Biodegradation of a hydrocarbon contamination by indigenous microorganisms is one of the cheapest contaminant removal options; however, it may take a long period of time, especially in extreme environments, such as non-optimal hot or cold air temperatures. Microorganisms present in the soils can adapt to sudden environmental changes, e.g., soil moisture changes, or changing energy sources such as after a hydrocarbon fuel spill. Some microorganisms can use the contaminant as their main carbon source; after acclimation to the changed conditions the natural degradation process begins [1]. The biodegradation process can be particularly slow if the surrounding conditions such as temperature, soil moisture content, or the type of soil are not favorable [2,3,4]. During biodegradation of hydrocarbons, aerobic microorganisms can metabolize organic compounds from the fuel spill as their energy source, producing CO_2_ and water [5]. Thus respiration rate measurements as CO_2_ production can be used as an indicator of aerobic microbial activities. The most easily degradable components of the hydrocarbon fuels are the intermediate-chained compounds, enabling rapid growth of the indigenous microbial communities [6]; therefore, they are an important component of the overall biodegradation process. However, as the easily degradable compounds from the fuel source are diminishing fast along with available nutrients from the surrounding soils [7], the biodegradation process can significantly slow down, and the less degradable compounds can remain in the soil for a prolonged period of time after the occurrence of the contamination event [2,8]. In hydrocarbon degradation experiments, the short-chained alkenes can be highly toxic to many microorganisms; however, they might also evaporate fast into the atmosphere [6] resulting in a change of contaminant concentrations and overall toxicity to the microorganisms in the soil.

Mycoremediation of hydrocarbons, especially soil polluted with petroleum-based hydrocarbons, has been the focus of innovative soil and air remediation strategies in recent years; however, the role of different fungal species in the degradation process is still not fully understood [9]. Fungal soil remediation is still less studied than bacterial hydrocarbon degradation processes [10]. Fungi might be more advantageous over bacteria in the remediation process as they do not depend on merely soluble, readily absorbed organic compounds for nutrition [11]. The use of fungi for air biofiltration has also been investigated in recent years [12]. Fungi possess metabolic abilities to assimilate mono-aromatic compounds and also they can survive in growth-limiting conditions such as low pH or moisture [12]. Many fungal species are identified as able to degrade volatile hydrocarbon compounds, such as the ascomycetes genus *Fusarium* [13]. Previous studies noted the importance of fungal and bacterial remediation [14]. Therefore it is important to investigate the relative differences between mycoremediation of different fuel types as done in the present study. This can provide valuable information on overall contaminant removal processes and governing factors.

In this research five fuel types were used: (1) Conventional diesel, (2) heating diesel (HTG), (3) arctic grade Syntroleum, (4) processed fish biodiesel (B100), and (5) a blend of 20% fish based biodiesel with conventional diesel (B20). The arctic grade Syntroleum fuel used here was produced from natural gas through the Fischer-Tropsch gas-to-liquid technique by the Syntroleum Corporation (Tulsa, OK, USA). Syntroleum fuel is less toxic than conventional diesel due to its minimal content of aromatics, sulfur, and heavy metals [15]; therefore it might pose less environmental hazard than diesel fuel.

Fish biodiesel processing became of interest in Alaska around 2004 as an option for lowering diesel fuel dependency by using a cheaply available alternative source. As a result of this initiative, Alaska has produced up to 13 million gallons of biodiesel a year [16,17]. Fish biodiesel might be used as a heating fuel in rural Alaskan communities, mixed with high sulfur diesel, which is commonly used as a heating fuel. Another potential use would be as a blend with diesel fuel for vehicles. Therefore in the present study both heating and conventional diesel fuels were investigated and compared with alternative fuels with respect to their biodegradability.

The main objective of this study was to investigate the biodegradation process of hydrocarbon fuels by indigenous microbial communities, and to further investigate the ability of fungi to degrade these different hydrocarbons including volatile hydrocarbon compounds. The present research investigated the growth of fungal communities on volatile compounds under minimized or soil-free environmental conditions. At the end of the study, most fungi that were capable of growing on different fuel contaminated soils were identified by using microscopy and DNA sequencing. We hypothesized that fungal degradation is highly dependent on the type of hydrocarbon fuels, and that animal (fish) based fuels can be degraded by mycoremediation more easily than crude oil based sources such as diesel fuel.

## 2. Materials and Methods

### 2.1. General Setup

For the first part of the experiment, 1 kg of soil (sand) was placed in an airtight 2.5 L cylindrical glass container with an internal diameter of approximately 11.7 cm, creating a surface area of 107.5 cm^2^, filled up to approximately 27% of their height of 24 cm. The sand originated from an interior-Alaskan river bed. Quantified amounts (2000 mg/kg dry soil) of the chosen contaminant, i.e., arctic grade Syntroleum, conventional diesel #2, heating diesel (HTG), fish biodiesel (B100), or biodiesel blend (B20) were added to the surface of previously uncontaminated soil. Control setups with no fuel addition were also performed. The control respiration values were used as a baseline and subtracted from the values measured for the fuel contaminated samples. Additionally, 1000 mg of soil with known existing microbial cultures from previous experiments, that were already adapted to diesel fuel hydrocarbons, was sprinkled over the top of the contaminated soil to provide an inoculum of microbes and shorten the microbial adaptation period to the contaminant [18]. Fertilizer of the type 20-20-20 (N-P_2_O_5_-K_2_O, where the total nitrogen ingredients were: 20% ammonia N, 30% nitrate N, and 50% urea nitrogen) was dissolved in 10 mL of distilled water with a final concentration of 30 mg N/mL of solution, and was scattered over the soil surface, achieving a nutrient concentration of 300 mg N/kg dry soil. The amount of nutrient to be added was determined in a previous study by Niemeyer and Schiewer [19], representing optimal nutrient concentrations for the biodegradation process. The soil was not mixed after or during these additions to better represent natural conditions after a spill on the soil surface. The samples were incubated at 20 °C and data were collected during a 28-day long period, which time frame enabled the investigation of the most active biodegradation period [1].

Fish biodiesel blends were prepared on the volume percent based method (ASTM D 6751-08, 2008).

### 2.2. Soil Analyses

General soil analysis for the soil type used in the first part of the experiments (Section 2.1) was performed using ASTM standards for sieving, gravimetric water content (GWC), soil porosity, and bulk density determination prior to the experimental setup. A Dionex DX 500 ion chromatograph with PeakNet software was used for extractable ionic nitrogen species analysis at the end of the experiment to determine nutrient utilization (Section 3.3). Additional analyses for nitrogen and carbon concentration were done using an elemental combustion system (Costech Instruments).

### 2.3. Experimental Setup for Fungal Growth on Volatiles

The second part of the experiment investigated fungal survival, growth and respiration under adverse conditions where moisture content was low and only volatile compounds in the headspace were available as a main carbon source. Fungal communities were grown with conditions similar to the first part of the experiment. The experimental setup involved the same type of 2.5 L airtight jars, but without soil. Two thousand mg of a specific fuel was placed at the bottom of the jar. Approximately 10 cm above the fuel, a net with an area of 15 cm^2^ was suspended to support the growth of fungal communities, such that only volatile hydrocarbon compounds were available for carbon source. Concurrently, control setups were implemented with no fuel added to the jars. Two different setups were used, one involved some soil particles collected with fungal hyphae and placed on the grid, generally less than 1000 mg; for the other no soil was present, only fungal hyphae were placed on the grid. This setup enabled collection of mycelium samples for determination of the fungal species without contamination by soil particles and bacteria therein. No water or nutrients were added during the first four weeks, creating conditions where the air humidity level was relatively low. The total length of the experiment was 41 days, with 10 mL water addition at the bottom of the jar at day 28 to increase air moisture levels to achieve more favorable conditions and enhance further fungal growth. Experiments were carried out in either duplicates or triplicates, with average deviations of 4.6% from the mean.

### 2.4. Respiration Measurement

Carbon dioxide is the main product generated when hydrocarbons are completely mineralized in aerobic degradation processes. The respiration rate, i.e., the rate of CO_2_ production, was measured as an indicator for microbial activity. Typically, the containers were opened once daily for re-aeration and to measure the evolved CO_2_, which had been captured in 20 mL of 1 N NaOH solution [9]. CO_2_ measurements were taken by using a method and formula developed by Stotzky [20]. The carbonate formed was precipitated with a 0.3 N BaCl_2_ solution.

To relate carbon dioxide production quantitatively to the degradation of hydrocarbons, the following generalized stoichiometric equation for CO_2_ production was used, assuming a molar C:H ratio of 1:2 for diesel fuel:

2 CH_2_ + 3 O_2_ → 2 CO_2_ + 2 H_2_O (1)

This stoichiometric equation applies only to mineralization of the fuel. The predominant importance of the added fuel compared to other possible substrates was confirmed by the use of controls without fuel addition. A baseline was developed based on uncontaminated soil samples for comparison with the calculated CO_2_ production of the degradation experiments.

### 2.5. Modeling

First-order degradation rate constants were determined by using the integral method based on the calculated amount of substrate still available according to respiration data,

*lnC_t_* = *lnC*_0_ − *k* × *t* (2)

where *C_t_* is the remaining contaminant concentration at time *t*; *C*_0_ is the initial contaminant concentration; and *k* is the rate constant. Since previous research [1] had shown that the first order model fit well if separate rate constants were determined for two or three phases; that approach was used here. The lengths of phases were determined by visual inspection of the first order plot and the rate constants for each phase were determined by nonlinear fitting, minimizing the root mean square error (RMSE) between model prediction and experimental data (Microsoft Excel Solver function).

### 2.6. GC-MS Analysis of Hydrocarbons in Soil

To evaluate the relationship between substrate use and CO_2_ production, gas chromatography–mass spectrometry (GC-MS) analysis was performed (Agilent Technologies 6890N Network GC System coupled to a 5873 mass selective detector with column parameters: 30 m by 250 μm by 0.25 μm) and compared to respiration data. For this purpose, the diesel range organics (DRO) determination was conducted using a modified method that was developed based on the AK 102 and EPA 8270 (semi volatile organics by GC-MS) methods as described by Horel and Schiewer [21]. Briefly, the GC–MS DRO method used a splitless injection with helium carrier gas (pressure: 6.895 kPa; flow: 0.5 mL min^−^^1^; average velocity: 26 cm s^−^^1^). The oven temperature started from 40 °C and increased till 320 °C for the duration of 52.33 runtime. Surrogate (p–terphenyl–d14 with a concentration of 4000 μg mL^−^^1^) was used to evaluate the extraction efficiency and internal standards (Perylene–d12 with a concentration of 5000 μg mL^−^^1^) were used to verify instrument reliability after being in use for a longer time period. Recovery data analysis was performed for determination of the actual hydrocarbon recovery value by the gas chromatography method used. For this purpose, the soil was spiked with a known amount of fuel and extracted immediately. Soil samples of 100 g (dry weight) were dried with anhydrous sodium chloride and extracted 3 times with 200 mL methylene chloride solvent. Solutions were then evaporated to a final volume of 2 mL prior to GC-MS analysis.

### 2.7. Identification of Fungal Species

Isolates and DNA extraction—twenty-four specimens were collected from several experimental setups from the first day up to four weeks after visual fungal growth was observed with fish biodiesel as substrate. DNA was extracted from small samples of fresh mycelia using the DNeasy Plant Mini Kit (QIAGEN, Inc., Valencia, CA, USA).

The entire internal transcribed spacer (ITS) + 5.8S ribosomal subunit gene region of the nuclear ribosomal DNA (rDNA) repeat was amplified using polymerase chain reaction (PCR) in reaction mixtures containing 1.75 µL Ultrapure Water (Invitrogen), 1 µL 10× Herculase PCR buffer (Stratagene), 0.05 µL 100 mM dNTP mixture, 25 mM of each dNTP (Applied Biosystems), 0.2 µL Herculase DNA polymerase (Stratagene), 2 µL of 1 µM forward primer, ITS1F [22] and reverse primer, ITS4 [23], and 3 µL of template DNA. PCR reactions were performed using the following temperature program: 95 °C/2 min, 34 cycles of 95 °C/0.5 min, 54 °C/1 min, 72 °C/2 min; and 72 °C/10 min. Amplification products were subjected to gel electrophoresis in a 1.5% agarose gel and stained with ethidium bromide for visualization of the bands. PCR products were purified using the QIAquick^®^ PCR Purification Kit (QIAGEN, Inc., Valencia, CA, USA). The purified amplification products were sequenced, with the same primers as in the PCR reactions, using the Applied Biosystems (ABI) BigDye v. 3.1 terminator kit and an ABI 3100 capillary DNA sequencer (Applied Biosystems, Foster City, CA, USA).

The sequences were aligned by Clustal_X2 (Windows, version 2.1). Sequences were then subjected to similarity searches using the Basic Local Alignment Search Tool (BLAST) algorithm at National Center for Biotechnology Information (NCBI) website to identify samples. A phylogenetic tree of fungi was constructed with the neighbor-joining algorithm [24] of Clustal W and viewed with Mega X software (Windows, version 10.0) [25]. The evolutionary distances were computed using the Maximum Composite Likelihood method [26] and are in the units of the number of base substitutions per site. This analysis involved 50 nucleotide sequences. Codon positions included were 1st+2nd+3rd+Noncoding. All ambiguous positions were removed for each sequence pair (pairwise deletion option). There were a total of 1732 positions in the final dataset. Evolutionary analyses were conducted in MEGA X [27].

### 2.8. Statistical Analysis

The statistical analysis used to compare treatments was a two tailed, paired, *t*-test and single factor ANOVA within the different treatment groups. When significant differences occurred, Tukey’s honestly significant difference (HSD) post hoc tests were used to determine significant interactions. The statistical significance in the analysis was defined at *p* < 0.05. Error is reported as standard error.

## 3. Results and Discussion

### 3.1. Soil Characteristics

The pH of the soil was analyzed before the experiments, using a Mettler Toledo pH meter at 21.8 °C. The soil pH value was between 7.07 and 7.25, which is optimal for hydrocarbon degradation [28,29]. Fungal communities have a higher range of acceptance towards more acidic conditions [30,31], which might be more applicable to field conditions as pristine rainwater has the pH less than 7 [32]. The original gravimetric water content before the start of the experiments was negligible, less than 1%. The soil particles were ranging mainly between coarse to very fine sand with less than 0.3% of clayey material. The average bulk density was between 1.4–1.6 g/cm^3^. The average porosity of the sand varied between 39.7 and 49.3% dependent on the amount of clay present in the soil. The average percent of available total nitrogen in the soil was 0.0054% and the fraction of total carbon was 0.165% with the C:N ratio of 185:6.

### 3.2. Hydrocarbon Degradation Rate Constants and Mass Balance for Different Fuels

Initial research showed that Syntroleum is more degradable than conventional diesel fuel due to its higher content of straight aliphatics (C8–28) [2,33], which are typically more easily degradable than branched or cyclic hydrocarbons [6]. Similar findings were also observed in the present study. Conventional diesel fuel has a higher amount of branched and cyclic hydrocarbons compared with Syntroleum, and consequently higher degradation rates were observed for the Syntroleum fuel. The highest degradation rate constants were measured for B100 with averaging *k* = 0.0258 during a 28-day period. Syntroleum and B20 contaminated soil samples had similar degradation rate constants (*k* = 0.0162 and *k* = 0.0166, respectively). The lowest rates were observed in the heating diesel contaminated samples (*k* = 0.0084), which could be attributed to the higher amount of sulfur in the fuel compared to conventional diesel; however, the difference between degradation rate constants for the two types of diesel fuel were relatively small (*k*_Diesel#2_ = 0.0105).

**Figure 1:**
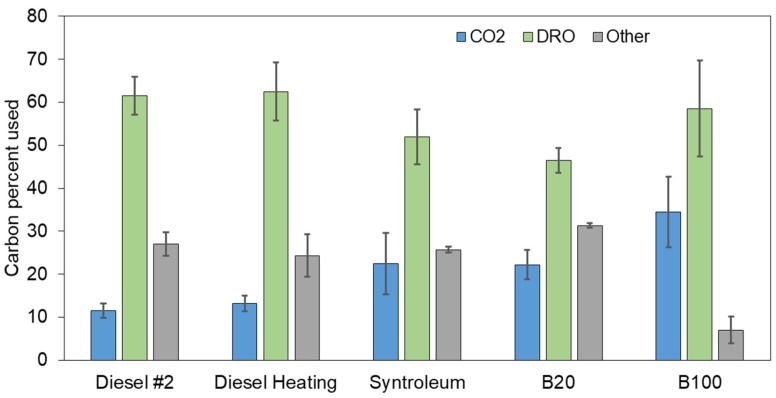
Percent carbon mass balance for different fuel types at the end of the experiment based on respiration (CO_2_) and Diesel Ranges Organics (DRO) measurements by GC-MS. Conditions: 20 °C; 300 mg N, 1 kg of sand; 2000 mg of diesel, heating diesel, Syntroleum, B20, and pure fish biodiesel (B100). Values represent means ± SE.

At the end of the experiment for most samples 69–75% of carbon originally present was recovered from respiration data and GC-MS analyses (Figure 1). The lowest hydrocarbon mineralization was observed for conventional diesel fuel contaminated samples (12%), the highest value for the B100 samples (34%). Significant differences were observed between Syntroleum, B20, and B100 compared to diesel fuel mineralization values (*p* < 0.001). There were no significant differences between Syntroleum and B20 samples (*p* = 0.33) and between diesel and heating diesel samples (*p* = 0.05). The total hydrocarbons remaining in the soil as DRO after four weeks were highest for diesel, which was over 57%. However, similar values were observed in all extracted samples (Figure 1). While volatilization was not included in the total carbon mass balance, it was estimated that up to 20% of mass-based changes can be due to volatilization in the case of diesel fuels and Syntroleum, while volatilization of B100 is assumed to be insignificant based on earlier studies [19]. Estimated biomass production could explain about half of the unaccounted carbons in the total carbon mass balance, as nitrogen use and carbon mineralization can be directly proportional to each other during the biodegradation process [14,21].

**Figure 2:**
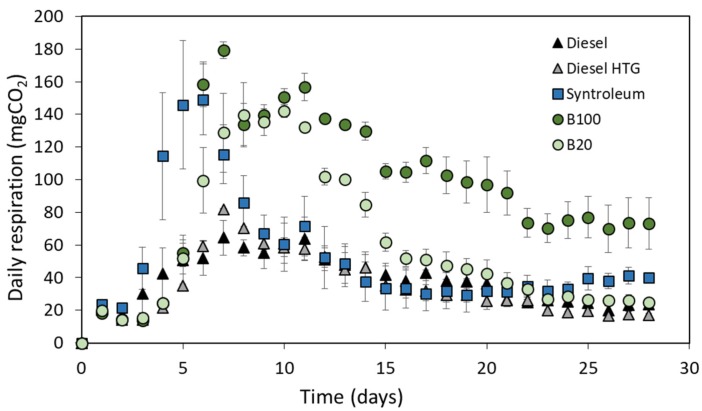
Daily respiration (CO_2_) values for conventional diesel, heating diesel (HTG), Syntroleum, B20, and B100. Conditions: 20 °C; 300 mg N, 1 kg sand; 2000 mg of selected fuel. Values represent means ± SE.

Intermediate-chained hydrocarbon alkenes were being used as main carbon source by the microorganisms after the brief initial lag phase period, and high microbial activity took place during a short period of time of less than six days as shown in Figure 2. The microbial respiration values showed that B100 had the highest average daily CO_2_ production (179.3 mgCO_2_; Figure 2). Syntroleum and B20 fuels also showed high daily CO_2_ respiration values (up to 149.1 and 141.9 mgCO_2_, respectively). B100 fuel degradation was consistently high after reaching the exponential phase of microbial growth, while all other fuels showed continuous decreases (Figure 2), as it is often the case in fresh fuel spill studies [34]. This phenomenon resulted in a significant overall fuel degradation and bacterial and/or fungal biomass production for B100 compared to the other fuels. In general, the lowest average daily respiration was observed in the case of diesel heating fuel (39.8 mgCO_2_/d), while the highest in the case of B100 (105.0 mgCO_2_/d) during the first 28 days of the fuel contamination (Figure 2). Biofuel biodegradability should greatly exceed the degradability of petroleum diesel fuel due to biofuels’ high fatty acid methyl ester (FAME) content [35]. Especially during the first few weeks of a hydrocarbon bioremediation process, the type of biofuel source, such as animal or vegetable based, can also influence the extent to which it is degraded [36]. The present study highlighted the fast degradability of the fish based fuel. Several studies found that the biodegradation of pure diesel fuel was much lower than the diesel/biodiesel blends, especially with an increasing amount of biofuel in the mixture [3,37]. Our findings also agree with these studies. In the present experiment, fish based biodiesel showed that 20% biodiesel addition to petroleum diesel fuel can increase daily respiration up to 78% compared to pure heating diesel and up to 41% when compared with regular diesel fuel.

### 3.3. Nutrient Availability and Utilization

The amount of nutrients in soil influences the effectiveness of overall bioremediation of hydrocarbons. Several cold region remediation studies have documented that nutrient deficient soil inhibits the biodegradation process [38,39]. The original nutrient amount was adjusted to 300 mg N per kg sand, based on earlier studies investigating similar environmental conditions for microbial degradation, such as diesel fuel contaminated subarctic soil [2,19]. Therefore, the present study used the optimal amount of available nitrogen for enabling fast microbial growth on the fuel source.

The amount of nitrogen use can be converted to an estimated production of biomass with the overall stoichiometric formula C_5_H_7_O_2_N.

(3) $$\%C\;\mathrm{converted}\;\mathrm{to}\;\mathrm{biomass}=\%N\;\mathrm{used}\;*\frac{300\mathrm{mg}N_{\mathrm{initial}}}{1700\mathrm{mg}C_{\mathrm{initial}}}\frac{60\mathrm{mg}C}{14\mathrm{mg}N_{\mathrm{biomass}}}$$

Nitrogen depletion from soil varied considerably between the different fuel types. For samples with fungal growth, relatively high nitrogen depletion was observed (up to 38.4%) compared to diesel degradation setups, where fungal growth could not be observed (12.0%). Based on nutrient depletion, diesel and heating diesel had low estimated biomass production of approximately 10%, while for biodiesel, where fungi were responsible for a significant amount of respiration, 16% of the initially present carbon may have been converted to biomass based on the above equation. This corresponds to observations of other researchers who noted that bacterial flora assimilates 5–10% of substrate carbon into new cells, while fungal flora assimilates 30–40% into new mycelium [40].

A C:N ratio above 9:1 is recommended for biodegradation of hydrocarbon fuels [41,42], below this amount the degradation by the indigenous microorganisms might be inhibited. While in the present study additional nitrogen was added to the soils, longer biodegradation processes might result in lowered nutrient availability for the microorganisms over time. Therefore, nitrogen deficient soils should be amended with readily available nitrogen sources for the microorganisms in the soil, such as ammonium based or urea nitrogen [38,43,44].

### 3.4. Degradation of Volatile Compounds by Fungal Communities

Previous research showed that removal of volatile hydrocarbon compounds from the headspace by activated carbon resulted in a considerable decrease of fungal colonization on the soil surface [21]. This finding indicated that fungi, which were able to utilize some volatile compounds as their carbon source, essentially contributed to degradation of biodiesel and its blends. Consequently, removal of volatiles by activated carbon had a large impact on biodiesel degradation by fungi. Therefore, to investigate fungal survival solely on volatile compounds, a separate experiment was carried out, where fungi were grown on a net suspended above the fuel with little (approximately 1000 mg) or no soil present.

**Figure 3:**
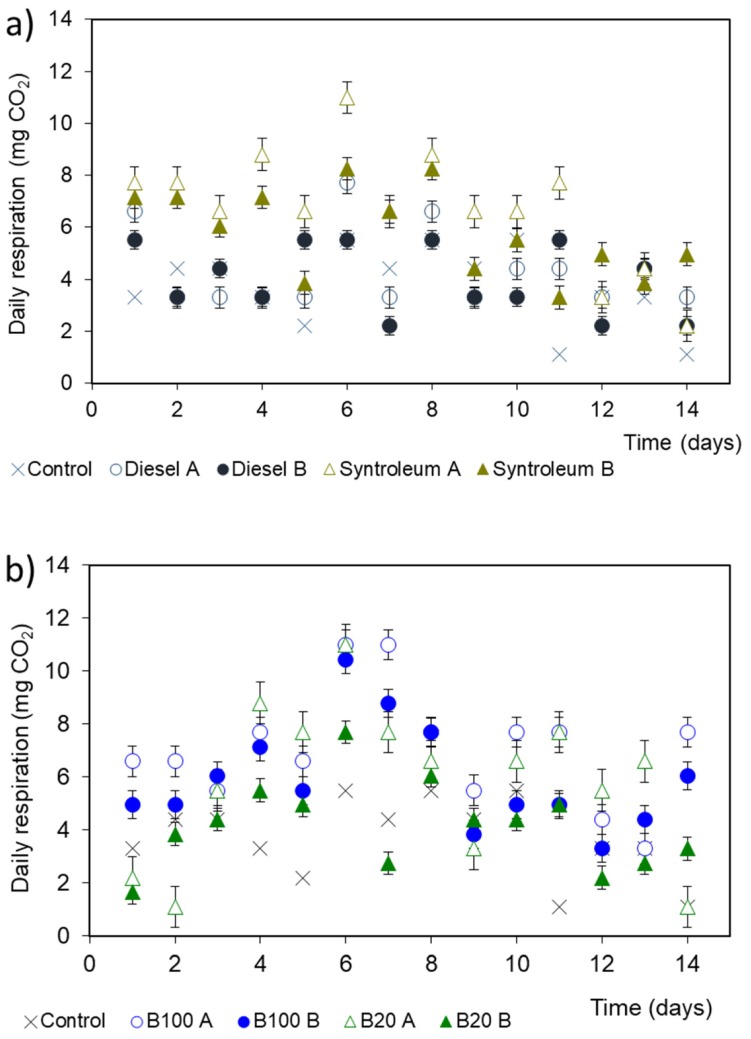
Daily fungal CO_2_ production based on volatile compounds for different fuel types. Conditions: 20 °C; ambient air humidity, 2000 mg of fuel placed without physical contact with soil or the investigated fungi. Symbol A represents experimental setups with ~1 g of soil, and symbol B without any soil. (**a**) Control, conventional diesel and Syntroleum; (**b**) control, fish biodiesel (B100) and fish biodiesel blend (B20). Values represent means ± standard error.

**Figure 4:**
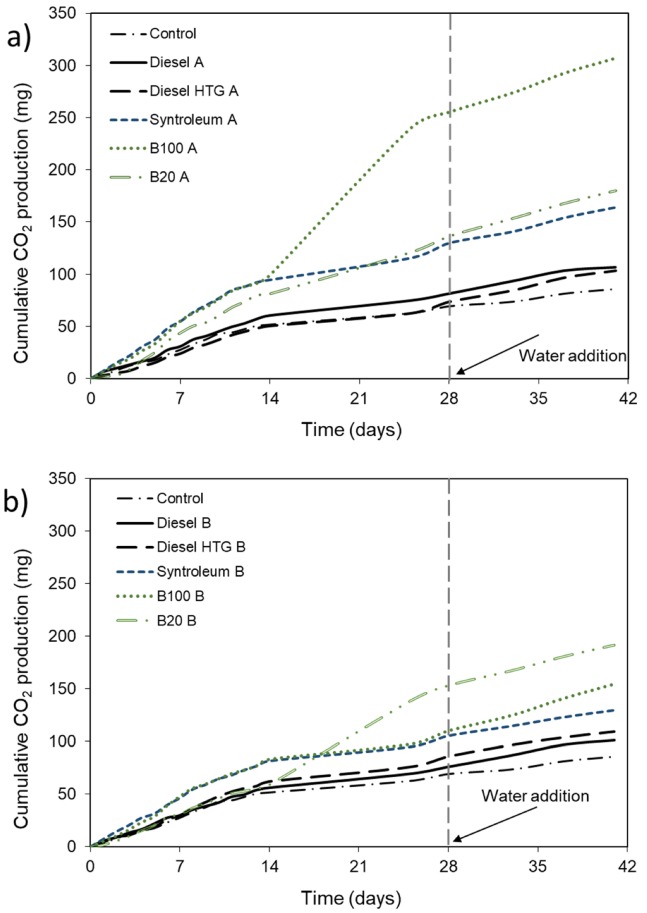
Cumulative fungal CO_2_ production based on volatile compounds for different fuel types. Conditions: 20 °C; control without fuel source, 2000 mg of conventional diesel, heating diesel fuel, Syntroleum, fish biodiesel (B100) or 20% fish biodiesel blend (B20) placed without physical contact with soil or fungi. (**a**) With ~1000 mg of soil (**b**) without soil. Ten mL of water was added after 28 days at ambient air moisture. Values represent means.

Fungal growth and respiration was observed in all experimental setups, which suggests that fungal fuel degradation can occur under non-favorable conditions. During the experimental time frame, CO_2_ production was much lower than in the first set of experiments where 1 kg of soil was present; however, low but measurable fungal respiration still occurred even after two weeks of non-favorable conditions (Figure 3 and Figure 4). Fungal communities in the experimental setups could not only survive on volatile compounds, which are toxic to most microorganisms [6], but also produced new hyphae. As expected, with some soil particles the daily respiration rates were higher than without soil, as shown in Figure 3 and Figure 4, especially for B100 and Syntroleum. As soon as water was added to the experimental space on day 28, the air moisture level increased and consequently the respiration rates amplified (Figure 4) to a cumulative 141 mg CO_2_ for the following 11 days (B100). While in the biodiesel treatments as much as 17 times higher daily respiration could be observed after the addition of water, in the heating diesel fuel treatments there was only approximately a five-fold increase after water addition. Statistically significant differences were only observed between the treatments with compared to without soil particles in the cases of diesel fuel and Syntroleum after moisture addition (*p* = 0.026 and *p* = 0.013, respectively), highlighting the requirement for water presence to adequate fungal hyphae growth and remediation progression, while for pure petroleum fuels bacterial density and diversity are the key factors. For B20 fuel, the experimental conditions (water addition, soil particles present) did not affect the respiration values significantly (*p* > 0.31).

Figure 4 shows the cumulative CO_2_ production for the different fuel types during the entire experimental duration. Interestingly, respiration for biodiesel and its 20% blend was highest, even though biodiesel showed the lowest amount of volatiles [21]. Though pure diesel and heating diesel had fairly low cumulative CO_2_ production, the overall amount was still substantial, with over 80 mg CO_2_ produced by day 28, before water addition, and over 100 mg CO_2_ produced by day 41. Most fuels showed fairly similar results varying between 103–180 mg CO_2_ with some soil particles (Figure 4a) and 85–157 mg CO_2_ without soil particles present (Figure 4b). Unusually high cumulative CO_2_ production by day 41 was observed for biodiesel (307 mg CO_2_) in the presence of some soil particles and B20 (191 mg CO_2_) without soil particles. The sudden increase in CO_2_ production around day 14 for these fuels was associated with a visible growth spurt of fungal communities in these two cases, indicating the importance of fungi in degradation of biodiesel and its blend.

The bioremediation of a single compound by a pure strain of microorganism can be studied more easily and such processes are better understood. However, hydrocarbon spills in natural environments involve diverse microbial communities degrading combinations of hydrocarbon compounds. Several studies demonstrated fungal isolates that grow on aromatic hydrocarbons [45], including BTEX [46], fungi using styrene from air [47], pyrene [48] or toluene [45,49] used as the sole source of carbon and energy for fungal growth. These results from literature and the one demonstrated in the present study, further emphasize the vital role of fungi in overall hydrocarbon bioremediation processes and the fungal ability to acclimate to adverse environmental conditions.

### 3.5. Identification of Fungal Species

Long-term environmental consequences of oil spills in soil could be less severe than for coastal ecosystems, mainly due to the presence of dense and diverse fungi and bacteria in the soil [50]. Most hydrocarbon degraders in soils are bacteria and fungi, which are generally abundant [31]. The cold-adapted microorganisms have modified enzymes with high catalytic activity in cold environments [51]; therefore microbial degradation under non-favorable conditions is still expected.

**Figure 5:**
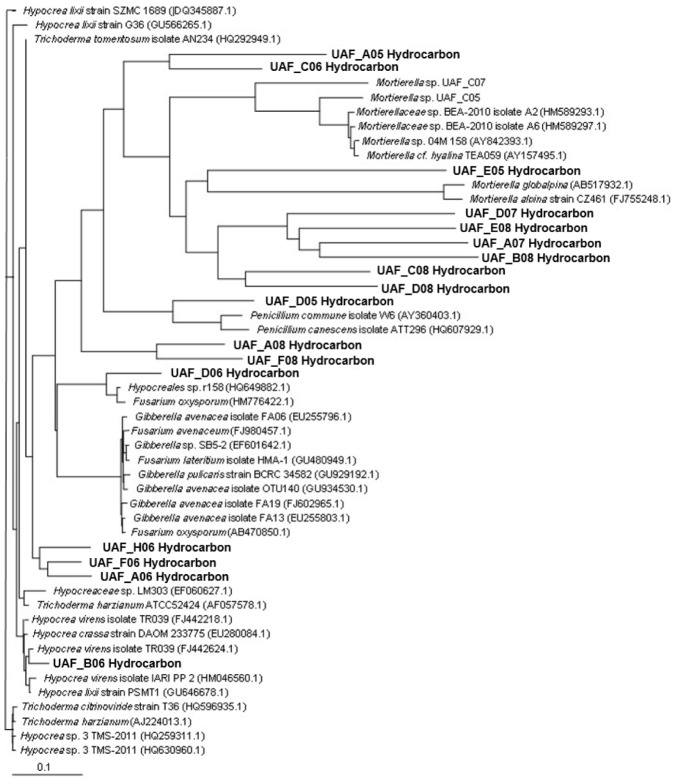
Phylogenetic tree of Syntroleum- and biodiesel-associated fungal species. The isolated species are shown in bold. GenBank accession numbers are in parentheses. Scale represents 0.1 substitutions per nucleotide position. The optimal tree with the sum of branch length = 5.75993432 is shown. The tree is drawn to scale, with branch lengths in the same units as those of the evolutionary distances used to infer the phylogenetic tree.

It was noted in prior research that fungal growth occurred on the soil surface using soil microcosms to study the biodegradability of fish biodiesel and Syntroleum [21]. For fish biodiesel samples, visual fungal growth could be observed by the fifth day of the present experiment with 1 kg of sand and for Syntroleum by the sixth day. The fastest fungal growth was observed in fish biodiesel contaminated soil samples. To identify the fungal species, additional experiments were performed with fish biodiesel contaminated soil without inocula, and samples were taken at different intervals from setups with fungal growth, i.e., on first day of visual fungal growth and when different types of mycelia were observed. These experiments were carried out for all fuel types; however, only soil with fish biodiesel and biodiesel blends as substrate showed visible fungal colonization and were consequently identified with DNA sequencing. In earlier experiments, fungal growth had also been observed for Syntroleum contaminated samples, some of which were used in the microscopic analyses, where *Trichoderma* was the main identified species for Syntroleum (Figures S1 and S2). The sequencing of the internal transcribed spacer (ITS) + 5.8S ribosomal subunit gene region was performed for all collected fungal samples and the results are summarized in the phylogenetic tree (Figure 5). Based on the similarity search (BLAST), the main species were *Hypocrea*/*Trichoderma* sp., *Penicillium* sp. *Giberella* sp., *Mortierella* sp., and *Fusarium* species. *Trichoderma harzinaum* and *Mortierella* sp. are reported as common fungal species of soil [31]. In general, all fish biodiesel or biodiesel blend contaminated samples showed fungal growth, which indicated mycoremediation as a major part of the overall biodegradation process for these fuels.

When diesel or heating diesel was utilized as the contaminating fuel type, mainly bacterial degradation was observed. Fungal communities did not appear in any of the experimental setups using diesel fuels. While in the present study the bacterial remediation might have outcompeted mycoremediation, it has been observed in other studies that *Aspergillus* and *Penicillium* species have the ability to grow and degrade crude oil [52], and bacterial and fungal co-cultures can improve hydrocarbon degradation [53,54]. April et al. [55] have reported that *Penicillium* and *Fusarium* species are petroleum hydrocarbon degraders in cold climates. In another study, *Trichoderma* sp. showed exceptionally fast growth and *Mortierella* sp. showed limited growth on diesel contaminated medium [56]. *Penicillium* and *Aspergillus* species have been frequently reported in the literature as filamentous fungi that grow on hydrocarbons [55,57,58]. While in the current experiment *Aspergillus* sp. could not be identified as one of the fungi present in the soils, this does not necessary indicate its absence. Husaini et al. [57] found that the highest average growth rates could be observed for *Trichoderma* species, which correlates with the current study based on visual observation. Moisture in the air and soil also enhances degradation by microorganisms, especially during mycoremediation, which was confirmed in the present experiment.

## 4. Conclusions

In this study, the biodegradation process was the fastest during the first few weeks of the remediation process. Fungal growths on contaminated soil were visible within one week of the experiments and produced a substantial amount of CO_2_, which continued to increase for a relatively longer period of time, especially for B100 contamination. Fungi present were identified as *Trichoderma* sp., *Penicillium* sp. *Giberella* sp., *Mortierella* sp., and *Fusarium* species. Fungi were able to grow suspended on a grid without any soil, solely using volatile compounds present in the air. Increased air moisture provided a more favorable environment for fungal growth, resulting in more complete fuel degradation. Based on our findings from the first experimental setup (using 1 kg soil), we found that, especially for B100 fuel, a combination of mycoremediation and bacterial remediation was advantageous over bacterial remediation alone, because the synergistic action prolonged the exponential growth phase, resulting in comprehensive mineralization of hydrocarbon fuel contaminants, especially for biodiesel fuels. The present study also observed that fungal colonies were able to use the volatile compounds from the different hydrocarbon fuels as energy source.

## Supplementary Materials

The following are available online at https://www.mdpi.com/2076-2607/8/2/163/s1, Figure S1. Microscopic pictures of a) fungal mycelium and b) *Trichoderma* sp. collected from fish biodiesel contaminated soil samples. Figure S2. Fungal growth on fish biodiesel contaminated soil sample.
