# Supplementary material for: Microbial Degradation of Different Hydrocarbon Fuels with Mycoremediation of Volatiles
Source: Microorganisms. 2020 Jan 23;8(2):163. doi: 10.3390/microorganisms8020163 (PMC7074729; doi:10.3390/microorganisms8020163)
Supplement: Supplementary file 1 [file microorganisms-08-00163-s001.pdf]

a)
b)

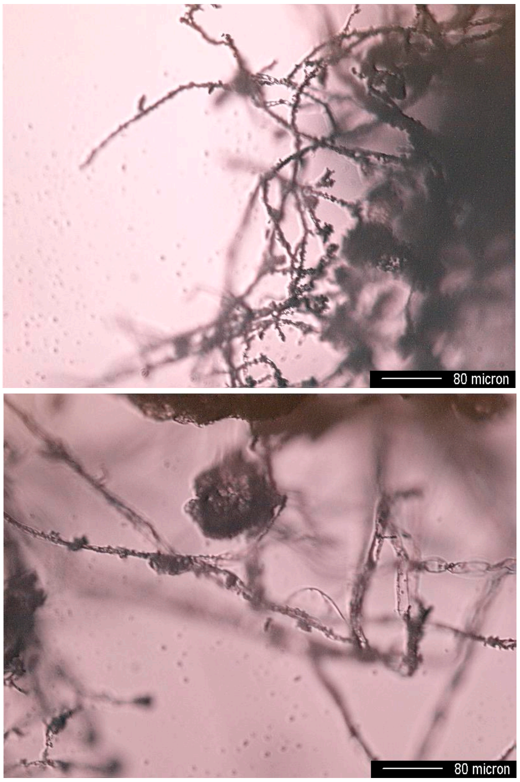

Figure S1. Microscopic pictures of a) fungal mycelium and b) *Trichoderma* sp. collected from fish biodiesel contaminated soil samples.

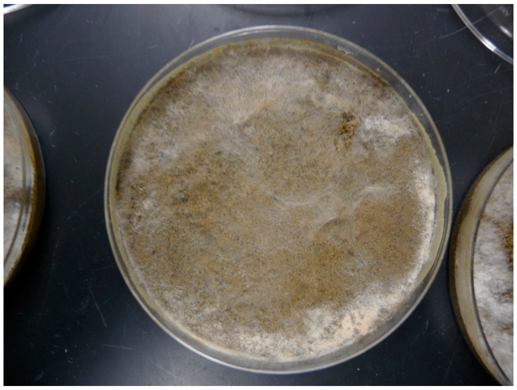

Figure S2. Fungal growth on fish biodiesel contaminated soil sample.
